# Management of Locked Volar Radio-ulnar Joint Dislocation

**DOI:** 10.1097/GOX.0000000000002480

**Published:** 2019-10-30

**Authors:** Marta Starnoni, Giulia Colzani, Giorgio De Santis, Andrea Leti Acciaro

**Affiliations:** From the * Division of Plastic Surgery, University of Modena and Reggio Emilia, Largo Pozzo 71, 41124 Modena, Italy; † Division of Plastic Surgery, University of Modena and Reggio Emilia, Largo Pozzo 71, 41124 Modena, Italy.

## Abstract

Isolated volar dislocation of the distal radio-ulnar joint is an extremely rare lesion. Diagnosis is commonly missed. The authors report their experience about a case of an acute locked volar distal radio-ulnar joint dislocation. A correct clinical and radiological diagnosis was done in the Emergency Department, and a closed reduction was achieved only after an axillary block, after a first failed attempt under slight sedation. A K-wire blocking the prono-supination and a short removable forearm cast protected the reduction for 25 days. Two weeks after the removal of the immobilization, the patient presented a complete functional recovery, with full range of motion. The authors highlight the importance of the clinical and radiological findings: a dorsal dimple at the ulnar side leads to a high index of suspicion, and represent the most relevant aid in diagnosis, associated to a proper imaging assessment. Prompt management allows a minimally invasive approach and a rapid functional recovery.

Volar dislocation of the distal radio-ulnar joint (DRUJ) is an extremely rare condition with only 35 cases reported in the literature.^1^ The most relevant aspect in the management of this injury is a rate of missed diagnosis up to 50%^2^ because of the sporadic presentation in the Emergency Departments, inducing lack of suspicion even in presence of evident clinical deformities. Moreover, X-rays might be often inconclusive due to incorrect execution and interpretation.^3,4^ In presence of a locked dislocation, a sudden closed reduction is the most appropriate treatment, which can be obtained under conscious sedation or by means of an axillary block.^3,5–7^ Only in case of a delayed diagnosis or persistent dislocation or instability the open reduction appears to be necessary.^8,9^

## CASE REPORT

**Fig. 1.**
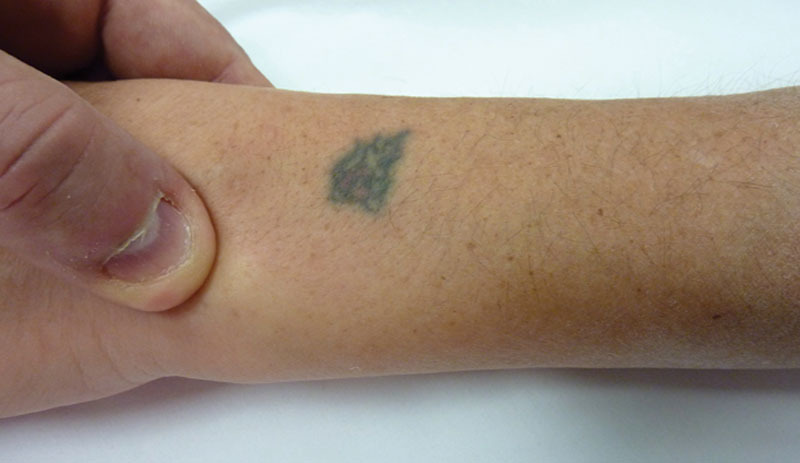
Clinical features showing the pathognomonic dimple at the dorsal ulnar side of the wrist.

**Fig. 2.**
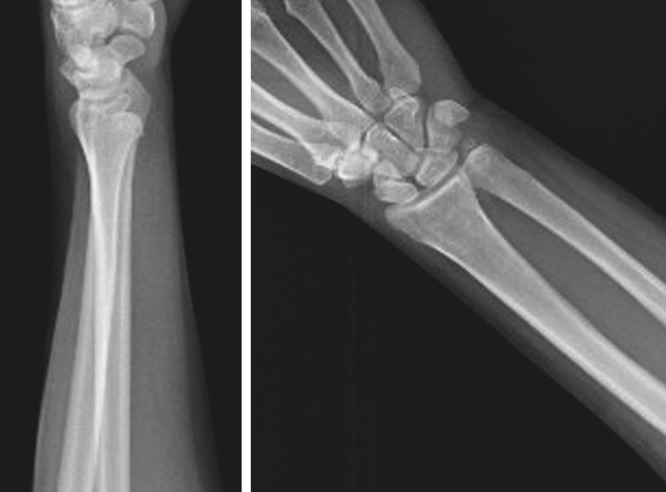
X-ray examinations. Antero-posterior and lateral views show the volar displacement of the ulna in relation to the DRUJ, even if the later view does not clearly show the dislocation, explaining the high index of suspicion necessary in this pathology.

A 42-year-old woman came to the Emergency Department presenting pain and functional impairment of the wrist that occurred during a Yoga posture. The traumatic mechanism was described as a hypersupination of the forearm with the wrist in a fixed position. The patient presented swelling at the wrist, particularly on the volar aspect. The absence of the dorsal ulnar head prominence was noted (Fig. 1). There were no neurological symptoms, the radio-carpal flexion-extension was preserved, the pronation of the forearm was painful and limited only in the extreme degrees. The X-rays in antero-posterior and true lateral view (Fig. 2) showed an anterior dislocation of the ulnar head with an associate fracture of the tip of the ulnar styloid, not involving the portion of insertion of the triangular fibrocartilage complex into the fovea. An attempt of reduction under slight sedation was performed unsuccessfully, so the patient was addressed to the Department of Hand Surgery considering the possibility of an open reduction.

**Fig. 3.**
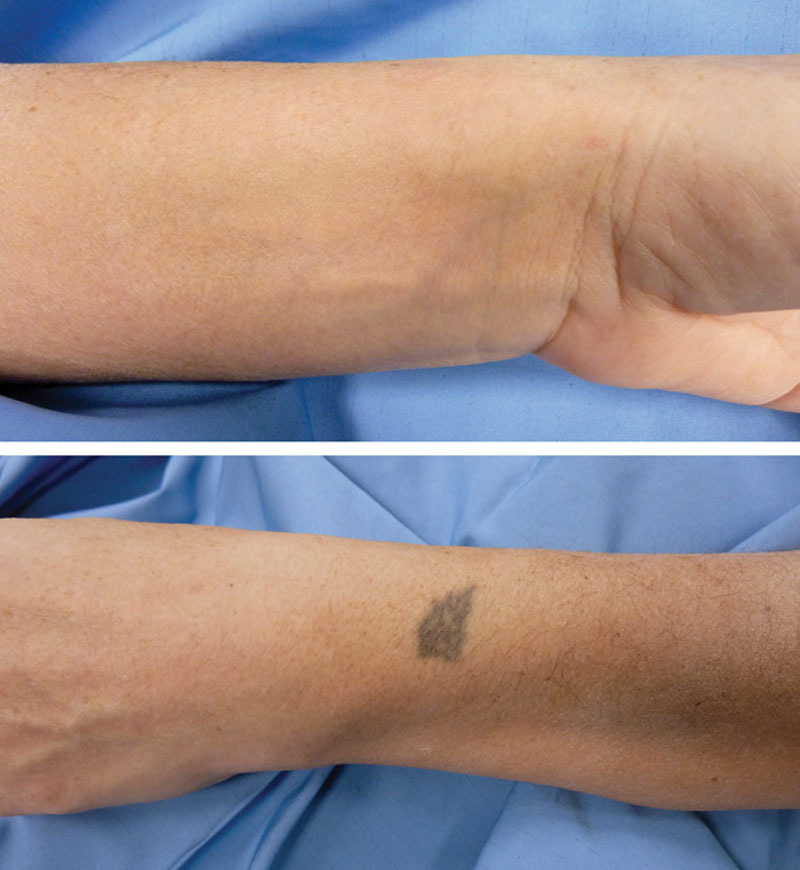
Clinical finding showing the correct reduction of the ulnar head, with respect to the preoperative dimple at the dorsal aspect of the wrist.

**Fig. 4.**
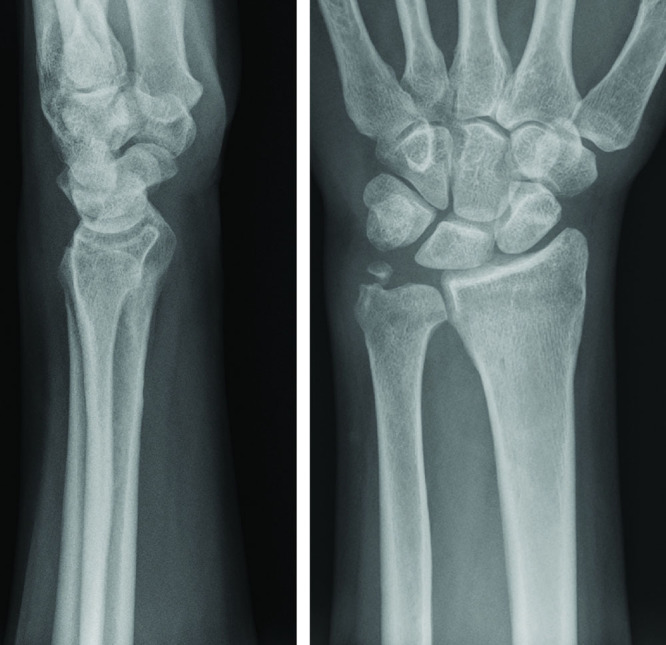
Antero-posterior and lateral X-ray follow-up examinations revealing the correct reduction and function of the RUD.

Nonetheless, under an axillary block, a closed reduction was obtained, performing a hyperpronation of the forearm with slight pressure directly on the volar aspect of the ulnar head, attaining the release of the contracted pronator quadratus muscle and avoiding excessive mechanical compression on the ulnar neurovascular bundle. Afterwards, the profile of the ulnar head at the dorsal aspect of the wrist was restored and the DRUJ was clinically stable (Fig. 3). The wrist was then fixed in slight pronation with a temporary K-wire and protected with a short removable forearm cast for 25 days. After reduction, a further stabilization by means of K-wires was added to decrease the chances of a new dislocation during the healing period and to be able to use a short plaster splint with more confidence. Subsequently the patient started a mobilization program according to the protocol provided by the Hand Rehabilitation Department. Two weeks after the removal of the immobilization, she presented a complete functional recovery with wrist and forearm full range of motion, without ulnar nerve impairment, pain, or residual instability at the DRUJ (Fig. 4).

## DISCUSSION

The DRUJ is the most relevant articulation involved in the rotational movements of the forearm. During supination, the ulnar head translates volarly, while during pronation dorsally. In the extreme degrees of rotation the sigmoid notch is no longer able to constrain the ulnar head.^3^ The hypersupination, exceeding also the protective action of the ligaments and the extensor carpi ulnaris,^9,10^ induces eventually the volar dislocation of the ulnar head, which tends to remain blocked because of the consequent spasm of the pronator quadratus.^3,6^ The presence of a dimple on the dorsal-ulnar side of the wrist after a trauma in hypersupination with the wrist in a fixed position represents the most relevant sign that must bring out the suspicion of a blocked volar dislocation of the ulnar head..^3,6^ However, pain and soft tissue edema made the clinical examination challenging.^3^ The antero-posterior and true lateral X-rays are crucial and mostly sufficient for diagnosis, but if not performed correctly (for example in case of malrotation of the forearm during the examination) could not show clearly the relationship between the bone structures and lead to misdiagnosis. Radiography is a first-level examination commonly used in the Emergency Department all over the world. It is of rapid execution and, if correctly performed and interpreted, is sufficient to arrive at a correct diagnosis in most of the cases, keeping in mind that a recognition and therefore a timely treatment lead to the best results. If X-rays are not diriment, a CT-scan may be useful.^5^ Magnetic Resonance can be performed to investigate the presence and entity of soft tissue injuries. Closed reduction by forceful hyperpronation of the wrist while pulling the ulnar head in dorsal direction under sedation is the treatment of choice in emergency, and surgery is required only when the dislocation persists or if a DRUJ instability remains. In literature, we found only one case in which the reduction was obtained also in hypersupination.^5^ After closed reduction, the DRUJ is generally stable, requiring 3–5 weeks of immobilization in an above elbow long cast with moderate up to full pronation.^3,5,6^ In our patient, we avoided the open reduction by means of a stronger analgesia through an axillary block, and the stabilization of the joint with a percutaneous K-wire allowed use of a short removable splint with the forearm in slight pronation for 3 weeks, permitting the flexion-extension of the elbow from the beginning and finally achieving the same good outcomes of the other reports in literature.

## CONCLUSIONS

An adequate knowledge of the biomechanics of the wrist and mechanisms of injury may increase the index of suspicion of these rare conditions, especially when the swelling masks the anatomical alterations.^2,4^ X-rays in two perpendicular projections are normally sufficient for diagnosis, if performed and interpreted correctly. Promptly closed reduction under appropriate analgesia is widely possible with a good residual stability of the DRUJ in most cases. In the light of all these considerations, we conclude that a correct recognition and management of such a condition, even if uncommon, is feasible in the Emergency Department also by inexperienced practitioner. Moreover, open surgery could be even more limited, considering that a closed reduction could be facilitated by a better muscular release obtained under an axillary block with a DRUJ sufficiently stable most of the times without the necessity of further invasive surgical gestures.
